# Mutation in Spike Protein Cleavage Site and Pathogenesis of Feline Coronavirus

**DOI:** 10.3201/eid1907.121094

**Published:** 2013-07

**Authors:** Beth N. Licitra, Jean K. Millet, Andrew D. Regan, Brian S. Hamilton, Vera D. Rinaldi, Gerald E. Duhamel, Gary R. Whittaker

**Affiliations:** Cornell University College of Veterinary Medicine, Ithaca, New York, USA

**Keywords:** Macrophage, pathology, protease, protein processing, feline coronavirus, viruses, feline enteric coronavirus, FECV, feline infectious peritonitis virus, FIPV, feline infectious peritonitis, FIP, conserved furin cleavage motif

## Abstract

Feline coronaviruses (FCoV) exist as 2 biotypes: feline enteric coronavirus (FECV) and feline infectious peritonitis virus (FIPV). FECV causes subclinical infections; FIPV causes feline infectious peritonitis (FIP), a systemic and fatal disease. It is thought that mutations in FECV enable infection of macrophages, causing FIP. However, the molecular basis for this biotype switch is unknown. We examined a furin cleavage site in the region between receptor-binding (S1) and fusion (S2) domains of the spike of serotype 1 FCoV. FECV sequences were compared with FIPV sequences. All FECVs had a conserved furin cleavage motif. For FIPV, there was a correlation with the disease and >1 substitution in the S1/S2 motif. Fluorogenic peptide assays confirmed that the substitutions modulate furin cleavage. We document a functionally relevant S1/S2 mutation that arises when FIP develops in a cat. These insights into FIP pathogenesis may be useful in development of diagnostic, prevention, and treatment measures against coronaviruses.

Feline infectious peritonitis (FIP) is a fatal infection that affects domestic and wild members of the family Felidae and is caused by a feline coronavirus (FCoV) of the family *Coronaviridae*, subfamily *Coronavirinae*, genus *Alphacoronavirus*, species *Alphacoronavirus*-1 (*1*). The FCoV genome is ≈29 kB and has 11 open reading frames encoding replicative, structural, and accessory proteins (*2*). Two serotypes have been identified. Serotype 1 FCoVs are highly prevalent clinically (*3*–*5*) but grow poorly in cell culture and are therefore underevaluated when compared with serotype 2 FCoVs, which are easily propagated in vitro but less prevalent. 

Within each serotype, there are 2 biotypes, each causing distinct disease outcomes. Feline enteric coronavirus (FECV) of serotypes 1 and 2 infects enterocytes, causing mild and generally self-limiting infections. FECV spreads efficiently through the oral–fecal route, and chronically infected cats can shed infectious virus in feces for a year or longer (*6*,*7*). The second biotype found in both serotypes, FIP virus (FIPV) is found less frequently but causes FIP.

The current understanding is that FIPV arises during in vivo infection from a genetic mutation of FECV (*8*–*11*). A long-standing hypothesis is that FIP viruses arise from internal mutation of endemic FECVs (*12*), which is believed to occur in approximately 1%–5% of enteric infections, resulting in the ability of the virus to infect blood monocytes and tissue macrophages. The resulting productive infection of these cells, a hallmark of FIP, enables systemic spread and results in macrophage activation, with concomitant immune-mediated events leading to death. To date, the precise mutation or mutations that cause a shift in FCoV biotype have not been identified.

As with other RNA viruses, coronavirus replication is error-prone; the estimated mutation rate is ≈4 × 10^−4^ nucleotide substitutions/site/year (*13*,*14*). It has been suggested that mutations in the 3c and 7b genes may be involved in the transition to FIPV (*1*,*12*,*15*). Because FCoV spike protein plays critical roles in receptor binding (S1) and fusion (S2), we focused on structural changes in this protein and potential role in altered cellular tropism. In particular, acquisition of macrophage tropism for a serotype 2 FCoV has previously been mapped to the spike gene (*16*), further suggesting that key mutations within spike protein may be important for the biotype switch.

The coronavirus spike protein is a class I fusion protein, which typically requires activation by cellular proteases. Mutation of the proteolytic cleavage site often has profound implications for disease progression (*17*,*18*). Until recently, FCoVs were thought to have uncleaved spike protein. However, a functional furin cleavage site has been identified in 2 serotype 1 FECVs, located at the shared boundary of the S1 and S2 subunits (*19*). Furin is a ubiquitous proprotein convertase enriched in the trans-Golgi network and is well-conserved among mammals (*20*). Furin cleaves a wide range of protein precursors into biologically active products at a consensus motif R-X-K/R-R, where R is the basic arginine residue, X is any residue, and K is the basic lysine residue (*21*).

In this article, we establish a novel approach to studying FIP that complements previous work. Instead of performing a mutation study based mainly on comparative genetic analysis (*15*,*22*–*24*), we focus on S1/S2, a functionally relevant site, and study variations between the biotypes and their functional effects. This rationale could provide a better means to uncover functionally important mutations that account for FIP.

We considered that mutations at the S1/S2 site could alter proteolytic cleavage and modify S fusogenic properties, leading to tropism expansion, systemic spread and, ultimately, FIP. We investigated genetic variations at the S1/S2 site of serotype 1 FECVs and compared these sequences to those present in viral RNA recovered from tissues of cats with FIP. Fluorogenic peptide cleavage assays were conducted to assess the effects of substitutions found in the S1/S2 site. We document a junction mutation at S1/S2 that arises during development of FIP. Our study has uncovered a molecular basis for FIP that has potential to lead to developments in diagnostics, prevention, and therapies.

## Materials and Methods

### FCoV Sequence Analysis 

Clinical and demographic data are reported in Technical Appendix Table 1). Fecal samples from asymptomatic infected domestic cats were solicited from shelters and veterinarians throughout the United States. RNA was extracted by using QIAamp Viral RNA Mini Kit (QIAGEN, Valencia, CA, USA). FCoV primers that detect most circulating strains were used to screen all fecal samples (*25*). RNA extracted from FIPV-TN406 (Black) laboratory-adapted strain was used as a positive control.

**Table 1:** Status of cats sampled for feline coronavirus and mutations in spike protein cleavage site*

	**FECV-infected cats**	**FIPV-infected cats**	**Total**
**Cats harboring viruses with >1 mutated S1/S2 site**	2	10	12
**Cats harboring viruses with an intact S1/S2 site**	28	1	29
**Total**	30	11	41

**FECV, feline enteric coronavirus; FIPV, feline infectious peritonitis; S1, receptor-binding domain of spike; S2, fusion domain of spike.**

We analyzed 22 FIPV-positive tissue samples (Veterinary Pathology Archives, Cornell University, Ithacan, NY, USA) from 11 cats with FIP. Diagnosis of FIP was based on the standard method of immunohistochemical evaluation by board-certified pathologists. Each sample was retrieved from formalin-fixed, paraffin-embedded tissue blocks from which sections were stained by using FIPV 3–70 antibody (Custom Monoclonals, Sacramento, CA, USA). Positively stained regions were thinly sectioned and RNA was extracted by using RecoverAll (Ambion, Foster City, CA, USA).

Fecal samples collected from FCoV-positive housemates, cats 234 and 304, were processed as previously described in this section. After the referring veterinarian made a diagnosis of FIP in cat 234, the owner elected to euthanize the animal. Fresh tissue was harvested and RNA extracted by using MagMAX Express (Life Technologies, Grand Island, NY, USA).

For all samples, 50 μL reverse transcription PCRs (RT-PCRs) were performed with One-Step RT-PCR (QIAGEN) by using gene-specific S primers, encompassing S1/S2. The PCR primers sequences are found in Technical Appendix Table 2. PCR conditions were 30 min at 50°C, 15 min at 95°C, and 39 or 35 cycles of 1 min at 94°C, 1 min at 55°C, 1 or 1.5 min at 72°C, and 10 min at 72°C. PCR products were purified by using a QIAquick Gel Extraction Kit (QIAGEN). Sanger sequencing was performed at the Life Sciences Core Laboratories (Cornell University). Nucleotide archive accession numbers are shown in Technical Appendix Table 4. DNA sequences were translated into protein sequences and alignments were performed by using Geneious 5.4 (Biomatters Ltd., Auckland, New Zealand). Sequence logos were generated by using Weblogo 3.1 (http://weblogo.threeplusone.com/). Statistical analysis was performed by using 2-tailed Fischer exact test. In the test, the numbers of FIPV-infected and FECV-infected cats were counted. For each category of FIPV or FECV infection, cats harboring viruses with or without mutations at the S1/S2 site were counted.

### Furin Cleavage A

### ssay

 Fluorogenic 12-mer peptides were designed and synthesized by RS Synthesis, Louisville, KS, USA (Technical Appendix Table 3). Purified recombinant human furin was purchased from NEB (Ipswich, MA, USA). For each reaction, 1 unit of enzyme was used in 100 μL final volume by using the reaction buffer 100 nmol/L HEPES, 0.5% Triton X-100, 1 mmol/L CaCl_2_, 1 mmol/L 2-mercaptoethanol, pH 7.5. Peptides were diluted to 50 μmmol/L. Reactions were performed in triplicate at 30°C and fluorescence was measured with a SpectraMax fluorometer (Molecular Devices, Sunnyvale, CA, USA), enabling Vmax determination. Results for each peptide are expressed as percent cleavage by furin compared with the canonical sequence.

To perform comparative analysis of the S1/S2 cleavage site between FECVs and FIPVs, we identified cases of FIP that were confirmed postmortem by using immunohistochemistry, the standard for FIP diagnosis; archival immunohistochemistry-positive formalin-fixed tissues were used as the source of FIPV RNA. To ensure good quality sequence information from archival material, the RT-PCR amplicon size was limited to 160bp (including the S1/S2 site). This same region was then amplified from fecal material from coronavirus-positive healthy cats.

## Results

### FECV S1/S2

**Figure 1:**
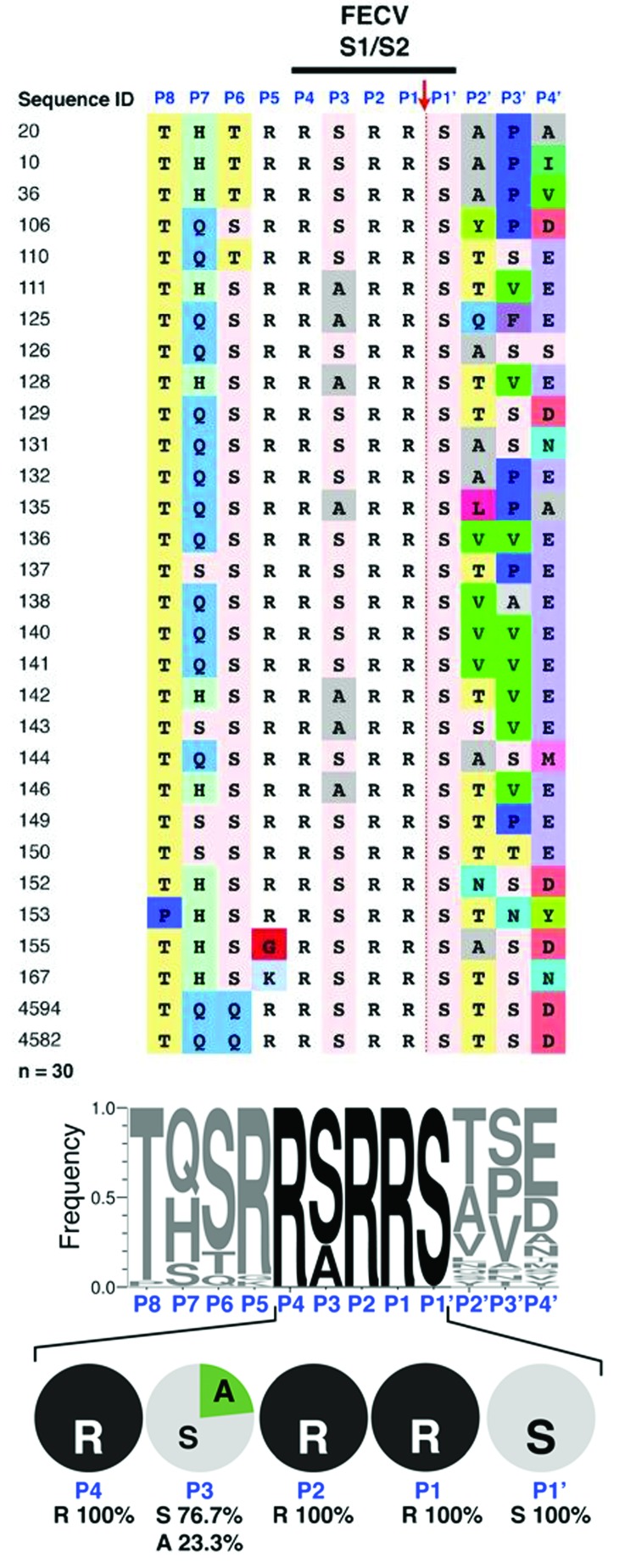
Sequence analysis of feline enteric coronavirus (FECV) spike S1/S2 site. RNA from 30 FECVs collected from 30 fecal samples obtained from subclinically infected cats was extracted, purified, and reverse-transcribed into cDNA. Sequencing of the spike gene was performed in a region surrounding the S1/S2 cleavage site. A) Sequence alignment. Sequence identification row (blue font): residue positions in the S1/S2 cleavage site from P8 to P4′. Red arrow indicates the site of furin cleavage. B) To visualize the diversity of residues at each position of the S1/S2 site, sequences were subjected to WebLogo 3.1 analysis (http://weblogo.threeplusone.com/create.cgi). Top: WebLogo for the 30 FECV S1/S2 sequences with the frequency of residue found at each position displayed. Bottom: summary of the diversity of residues for each position from P4 to P1′ and percentages of each amino acid represented.

**Figure 2:**
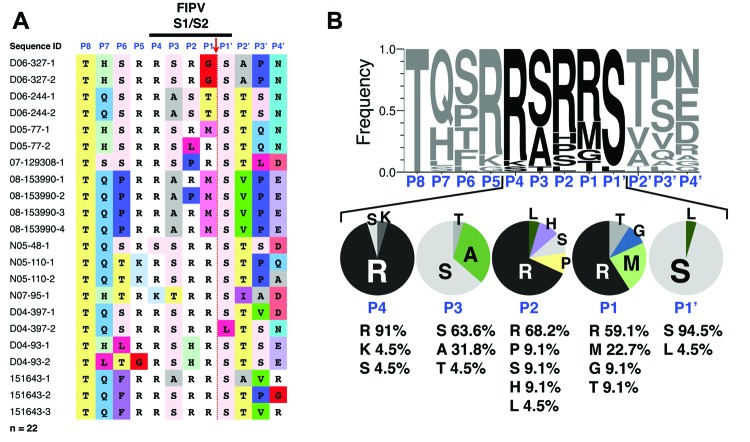
Sequence analysis of feline infectious peritonitis virus (FIPV) spike S1/S2 site. RNA from 22 FIPVs collected from 11 cats who had feline infectious peritonitis was extracted, purified, and reverse-transcribed into cDNA. Sequencing of the spike gene was performed in a region surrounding the S1/S2 cleavage site. A) Sequence alignment. Sequence identification row (blue font): residue positions in the S1/S2 cleavage site from P8 to P4′. Red arrow indicates the site of furin cleavage. B) To visualize the diversity of residues at each position of the S1/S2 site, sequences were subjected to WebLogo 3.1 analysis (http://weblogo.threeplusone.com/create.cgi). Top: WebLogo for the 22 FIPV S1/S2 sequences with the frequency of residue found at each position displayed. Bottom: summary of the diversity of residues for each position from P4 to P1′ and percentages of each amino acid represented.

Sequencing of the S1/S2 site of 30 S sequences from FECV fecal samples revealed an extremely well-conserved motif at the amino acid level (Figure 1, panel A). In particular, arginine (R) residues are found exclusively at the most critical positions for furin recognition and cleavage (P1, P2, and P4) in all sequences analyzed (Figure 2, panel B). The P1′ position is extremely well conserved, because serine (S) is found in 100% of cases. The P5 position is also well conserved, evidenced by a clear majority of basic residues found (96.6% arginine or lysine [K]; Figure 2, panel B). At P3, limited variability is found (76.7% serine and 23.3% alanine [A]). Overall, 100% of FECV sequences analyzed contain the furin cleavage site, with a core motif of R-R-S/A-R-R-S.

### FIPV S1/S2 

**Figure 3:**
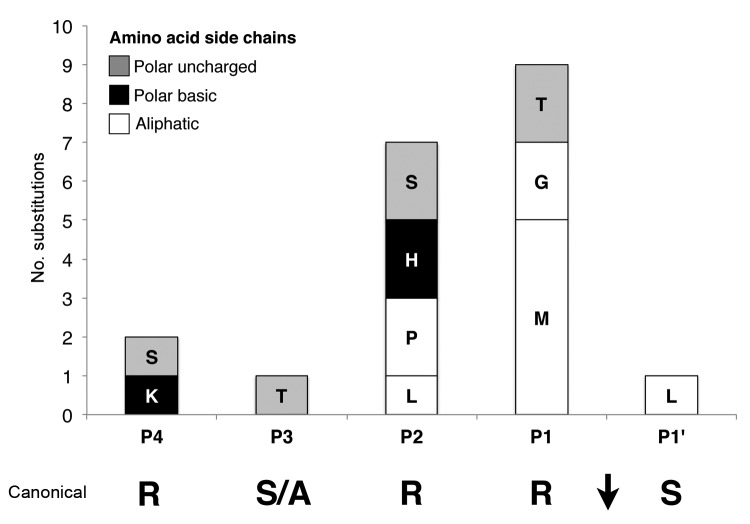
Amino acid substitution frequency at each position of the feline infectious peritonitis virus S1/S2 cleavage site. The histogram is based on feline infectious peritonitis virus S1/S2 WebLogo 3.1 analysis (http://weblogo.threeplusone.com/create.cgi), showing percentage of modification of residues at each position of the S1/S2 site, compared with feline enteric coronavirus S1/S2 canonical sequence consensus.

Analysis of the S1/S2 cleavage site of FIPV sequences shows that it has much more variability, both within the narrow furin cleavage recognition motif (P4-P1) and in residues extending out of it (P8-P5 and P2′-P4′) (Figure 2A). A striking observation is that the critical positions P1 and P2 are among the most consistently mutated (Figure 3). To a lesser degree, variability extends to other positions of the cleavage motif, notably in the P1′, P3, P4, and P5 positions (Figure 2). Examination results of the entire portion of spike sequenced in this study indicate that the conserved R-R-S/A-R-R-S motif in FECV is present within a region of the spike gene that shows a high degree of variability, in contrast to other neighboring regions that are more highly conserved (Technical Appendix Figure 1).

### Correlation between FIP Status of Cats and Presence of Mutations at S1/S2 

#### Fluorogenic Peptide Furin Cleavage Assay

**Figure 4:**
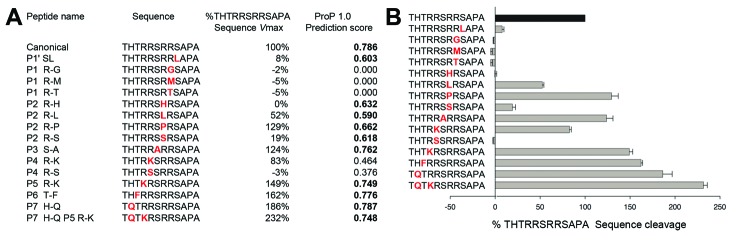
Furin cleavage assays of fluorogenic peptides. A) Synthetic fluorogenic peptides were generated with sequences matching consensus feline enteric coronavirus and a panel of modified sequences with substitutions (shown in red) found by feline infectious peritonitis virus sequencing. Peptides (50 μmol/L) were subjected to cleavage by recombinant human furin (1 U/100 μL), at pH 7.5, 30°C, and the release of fluorescence over time was measured by a spectrofluorometer enabling calculation of the Vmax of each reaction. Peptide cleavage scores generated by the ProP 1.0 server (www.cbs.dtu.dk/services/ProP/) are also displayed. B) For each modified peptide (substitutions shown in red), the percentage of cleavage rate compared with the canonical sequence was calculated and displayed. Cleavage assays were performed in >3 independent experiments. Error bars indicate SD for each measurement.

To test whether the identified FIPV S1/S2 mutations have an effect on cleavability by furin, we performed an in vitro proteolytic assay. We used human furin for these experiments. Human and feline furin are very similar (96% identical) and are expected to cleave in an equivalent manner. However, feline furin has not been directly studied to any degree, and reagents are not readily available. Feline and human cells lines show identical rates of cleavage for a known furin target protein (PSCK-9), which contains an active furin cleavage site (Technical Appendix Figure 2). We used fluorogenic peptides containing the canonical motif (R-R-S-R-R-S) or with substitutions from positions P1′ through P7 (Figure 4, panel A). The canonical peptide was efficiently cleaved by furin (Figure 4), with average Vmax of 235 Relative Fluorescence Units (RFU) per minute.

Within the P4-P1′ core peptide, in the canonical background, when the P1′ serine residue is changed into a leucine (L), furin cleavage is severely diminished (8% of canonical cleavage rate), a result that shows the key role of the conserved P1′ serine. Modifications of the P1 arginine in the canonical peptide, regardless of the residue tested, for example, glycine (G), methionine (M) or threonine (T), abrogate cleavage by furin (Figure 4). Modifications at the P2 arginine residue in the canonical peptide have variable effects. When P2 arginine is changed to histidine (H), there is complete inhibition (0% of canonical cleavage). When P2 is changed to leucine or serine, cleavage efficiency is reduced by ≈50% and 20%, respectively. When P2 is modified to proline (P), cleavage efficiency slightly increases to 129% of the canonical peptide (Figure 4). The P3 S-A substitution minimally enhances cleavage (Figure 4). P4 arginine is another residue position that is essential for furin cleavage. In the canonical peptide, P4 R-K substitution, there is a slight decrease in cleavage efficiency (88.7% of canonical rate). In contrast, when the P4 arginine is substituted with glycine, furin cleavage is completely abrogated (Figure 4).

For positions upstream of P4, while P5 R-K and P6 T-F modifications have moderate enhancing effects on furin cleavage (149% and 162% of canonical rate, respectively), the P7 H-Q peptide shows a substantial increase in its cleavability (186% compared with canonical). The P7 H-Q P5 R-K peptide shows that the effect of each modification can be additive (232% compared with canonical peptide) (Figure 4).

### Functionally Relevant S1/S2 Mutation

To further confirm our findings, we analyzed the S1/S2 sites from viral samples taken from cats 234 and 304, who lived in the same household (Table 2). At the initial sampling in 2009 (t = 1), both cats were asymptomatic for FIP and were shedding FCoV in their feces. In samples from both cats, the S1/S2 sites had a core sequence R-R-S-R-R-S consistent with the FECV consensus. Upon the second sampling in 2011/2012 (t = 2), FIP was diagnosed in cat 234. Cat 304 remained asymptomatic but continued to shed virus in feces. Notably, when the S1/S2 sequences were analyzed at the second sampling, only the cat with FIP (234) had a change in the FECV consensus sequence (a P2 R-L mutation). While exhibiting a change in the P3 residue (S-A), the virus present in cat 304 retained the conserved S1/S2 furin cleavage motif (Table 2). These data provide direct evidence of mutations in spike linked with development of FIP in cats.

**Table 2:** Sequence of FCoV spike at S1/S2 junction in cats sampled for feline coronavirus*

Time	Cat 234	Cat 304
t = 1	NHTHTRRSRR↓SAPVAV	NHTHTRRSRR↓SAPVAV
t = 2	NHTHTRRS**L**R↓SAPVAV	NHTHTRRARR↓SAPVAV

*Underlines indicate nucleotide substitution. FCoV, feline coronavirus; S1/S2, cleavage site of spike protein; t = 1, both cats disease-free; ↓ , cleavage site; t = 2, cat 234 feline infectious peritonitis–positive, cat 304 disease-free.

## Discussion

To study FIP, we have taken an alternative approach that complements earlier studies that were based on analytical outcomes of putative FIP-causing mutations and inference of their functional consequences. We focused on the S1/S2 sequence, a specific and functionally highly relevant cleavage site within the S protein, and documented mutations between asymptomatic and highly symptomatic cats that correlated strongly with FIP. We also documented a functional S1/S2 cleavage site mutation that arose in an asymptomatic cat that subsequently developed FIP.

Our sequence data show that serotype 1 FECV from feces of asymptomatic cats contain a highly conserved furin cleavage motif at the S1/S2 site, with the following narrow range of residues: (R>>K/G)^P5^-(R)^P4^-(S>A)^P3^-(R)^P2^-(R)^P1^-(S)^P1′^. In addition to the consensus R-X-K/R-R motif, additional flanking residues can also be consequential for furin-mediated cleavage (*26*–*28*). In particular, a serine (S) residue is critical in the P1′ position (*29*) and it is notable that all FECVs examined contained a P1′ S residue. The fact that the S1/S2 site is extremely well conserved is an indication that it is functionally essential for FECV replication in the enteric epithelium.

In contrast to the situation for asymptomatic cats infected with FECV, we found that sequences of FCoV sampled from tissue of confirmed FIP-positive cats consistently have mutations at the S1/S2 site. In the most critical position for furin cleavage, P1, we found that >40% of FIPVs have a mutation in the arginine residue, which is replaced by an aliphatic (methionine and glycine) or polar uncharged (threonine) residue. Overall, the distinguishing feature of FIPVs is the absence of the P1 arginine, rather that the presence of any particular residue. This is corroborated by our peptide cleavage data that demonstrate that furin cleavage is fully abrogated for all P1 substitutions tested. The next most common position mutated in FIPV is P2; >30% of the FIPVs analyzed bore mutations at this position. Most mutated residues found were aliphatic (P and L). Some sequences were substituted with a polar basic (H) or a polar uncharged (S) residue. Apart from the P2 R-P substitution, peptide cleavage data indicates that all other substitutions have an inhibiting effect on furin cleavage. Of note, for murine hepatitis virus (MHV), a betacoronavirus that also harbors an S1/S2 cleavage site in its spike protein, there is a precedent for the inhibitory effect of the introduction of a histidine in the P2 position of the cleavage site. Two well-studied strains, MHV strain A59 (MHV-A59) and the neurovirulent MHV strain JHM (MHV-JHM), have a notable difference at this site. MHV-A59 has an R-R-S-H-R-S sequence and is less efficiently cleaved than MHV-JHM, which has an R-R-A-S-S-R sequence (*18*). P4 is generally considered to be critical for furin cleavage, but we found limited variation in this residue position for the FIPVs tested and found mutation to the polar basic residue (K) or polar uncharged residue (S) in <5% of viruses. The peptide data indicates that, although introduction of a serine at P4 completely abrogates cleavage, the P4 R→K substitution has minimal effect. The FIPV P3 position showed small variation compared with FECV after the introduction of a polar uncharged residue (T) in 1 sample. For the P5 position, the only change was a slightly higher frequency of the lysine residue in samples from cats with FIPV. Peptide cleavage data indicated that the common S-A substitution found for FECV and FIPV P3 positions has only slightly increasing effect on proteolysis by furin. Furthermore, the P5 R→K substitution has an enhancing effect in the peptide cleavage assay. At P1′, the conserved polar uncharged residue (S) was retained in the majority of FIPV samples, however, the introduction of an aliphatic amino acid (L) was found. It is notable that furin cleavage has been suggested to be incompatible with a hydrophobic aliphatic side chain, with a strong preference for serine in the P1′ position (*27*,*29*). In the D04-397-2 sample containing the P1′ L, the basic residues within the S1/S2 site remain identical to the ones found in FECV sequence; we suggest that disruption of furin cleavage is mediated by a mutation in P1′, rather than the more typical P1, P2 and/or P4 mutation. This hypothesis is supported by the peptide cleavage assay, where the P1′S-L peptide is unable to be cleaved by furin.

Overall, in terms of FIP-positive animals, we found that 10 of 11 cats harbored viruses with mutations in the furin motif R-R-S/A-R-R-S found in FECV of asymptomatic cats. For most FIP-positive cats, we sequenced viral RNA collected from different tissues (Technical Appendix Table 1). Our data provides strong support for the internal mutation hypothesis, as the mutations are unique to individual cats. 

Of note, not all tissues from the same animal carry the same mutation. In some instances, mixed populations of viruses exist within the same animal. The majority of viruses sequenced had 1 mutation, although 5 (D06-244-1, D06-244-2, 08-153990-2, N07-95-1, and D04-93-2) had 2 mutations. However, there are 2 apparent exceptions of cats harboring viruses that do not have clearly defined mutations in the furin cleavage site: samples from cat 151643 (*1*–*3*) and samples from cat N05-110 (*1*,*2*). We consider that the presence of a P6 furin cleavage in samples of cat 151643 is consistent with our hypothesis of a switch in the activating protease for the virus, because this is not typical of naturally occurring furin cleavage sites. Samples 1 and 2 from cat N05-110 harbor virus with an atypical lysine residue at P5. While unusual for FECVs, a P5 lysine residue does appear to be compatible with furin cleavage, so it remains to be determined how noteworthy a P5 lysine residue versus a P5 arginine residue is in the context of a protease switch for FIPV, or whether other mutations correlated with FIPV in the case of this cat.

As part of our study, we analyzed field samples from cats harboring FCoV at different times. In cat 234, the virus underwent a transition from FECV to FIPV, and had a functionally relevant mutation in the S1/S2 motif (P2 R-L). Cat 304, living in the same house as cat 234, remained asymptomatic. Cat 304 harbored a mutated virus, but the mutation was in a functionally irrelevant position (P3 S-A). Identification of cats with FECV in which FIP subsequently develops is challenging, and while we present a single example, we consider these data to be strong evidence that mutations at the S1/S2 site are linked to a change in the pathogenic properties of the virus, and likely to be essential for the acquisition of macrophage tropism seen in FIP.

The S1/S2 cleavage site and surrounding residues of serotype 1 FIPV S sequences were found to be systematically modified by mutations. Chang et al. recently published an extensive comparative analysis of FIP mutations at the nucleotide level by performing whole-genome sequencing of FECVs and FIPVs; the authors found a site within S (nucleotide position 23531), but outside of S1/S2, to be the most frequently mutated in FIPV (*15*). We have undertaken an analysis of the S1/S2 sites sequenced by Chang et al. and find that our hypothesis that mutation within the S1/S2 furin motif correlates with FIP in ≈64% of their samples. There are 3 differences in methodology that may explain this lack of agreement: first, we employed immunohistochemistry to confirm the diagnosis of FIP, while Chang et al. reported using postmortem examination; second, all FIP samples in this study originate from tissue, while Chang et al. included both tissue and ascites fluid; and finally, we report multiple sequences for FIP-affected cats, while Chang et al. reported a single sequence. Sequence data from samples D04-397-1 and D04-397-2 provide evidence that both FECV and FIPV populations can be identified within an affected animal. Sequence information from a single sample may not be adequate for the detection of mutated virus. 

Most mutations negatively affect furin processing, but some enhance it. Given that the majority of the FIPV S proteins still harbor basic residues at the S1/S2 boundary, it could be reasoned that the mutated site becomes more open and can be cleaved by a range of other proteases. The switch in proteolytic requirements of S that we propose may offer an explanation for the crucial tropism transition during FIP. A possible consequence of the mutations is cleavability by monocytic/macrophage-specific proteases. These could be pro-protein convertases, cathepsins, or other macrophage-specific proteases. In particular, cathepsin B, matrix metalloproteases, and furin-related PCSK1 are likely to be expressed on the surface of macrophages and recognize the hallmark residues remaining or acquired in FIPV S1/S2 cleavage site (*30*). Matrix metalloprotease 9 is of particular interest because it was demonstrated to be upregulated in activated monocytes and macrophages during FIP (*31*). A shift in the entry pathway to enable virus entry at the cell surface instead of the endosome may simultaneously explain the ability of FIPV to infect macrophages and the macrophage resistance of FECV. It is also possible that the mutations in the S1/S2 region affect the heparin sulfate binding site in this region (*19*). However, heparin sulfate binding is a cell culture adaptation of the virus, and as so, its relevance to the clinical situation would appear to be unlikely.

A contrasting view to the internal mutation hypothesis to explain the genesis of FIP outbreaks is that there is a circulating FCOV other than FECV that is specific for FIP (*22*). For a complex disease process such as FIP, we and others consider it likely that there may be circulating FECVs that are closer to making the critical mutations necessary for FIP, possibly explaining paradoxical FIP outbreaks (*32*). Based on the data we present here, we conclude that mutation of the S1/S2 locus and modulation of a furin recognition site normally present in the S gene of FECVs is a critical contributing factor for development of FIP. Further studies could serve to analyze how S1/S2 mutations fit with the other mutations posited to account for FIP development.

Technical Appendix Clinical and demographic data for cats studied; coronavirus detection methods and materials.
