# Supplementary material for: Mutation in Spike Protein Cleavage Site and Pathogenesis of Feline Coronavirus
Source: Emerg Infect Dis. 2013 Jul;19(7):1066–73. doi: 10.3201/eid1907.121094 (PMC3713968; doi:10.3201/eid1907.121094)
Supplement: Technical Appendix — Clinical and demographic data for cats studied; coronavirus detection methods and materials. [file 12-1094-Techapp-s1.pdf]

Article DOI: http://dx.doi.org/10.3201/eid1907.121094

Mutation in Spike Protein Cleavage Site and Pathogenesis of Feline Coronavirus

Technical Appendix

Technical Appendix Table 1. Clinical and demographic data from 44 domestic cats sampled in the continental United States from 2004–2012

Cat no.	Sample no.	FECV/FIPV	Sample Type	Year	State	Sequence
20	-	FECV	Feces	2008	PA	THTRRSRRSAPA
10	-	FECV	Feces	2008	PA	THTRRSRRSAPI
36	-	FECV	Feces	2008	PA	THTRRSRRSAPV
106	-	FECV	Feces	2010	CT	TQSRRSRRSYPD
110	-	FECV	Feces	2010	CT	TQTRRSRRSTSE
111	-	FECV	Feces	2010	CT	THSRRARRSTVE
125	-	FECV	Feces	2010	WI	TQSRRARRSQFE
126	-	FECV	Feces	2010	WI	TQSRRSRRSASS
128	-	FECV	Feces	2010	WI	THSRRARRSTVE
129	-	FECV	Feces	2010	WI	TQSRRSRRSTSD
131	-	FECV	Feces	2010	WI	TQSRRSRRSASN
132	-	FECV	Feces	2010	WI	TQSRRSRRSAPE
135	-	FECV	Feces	2010	IA	TQSRRARRSLPA
136	-	FECV	Feces	2010	IA	TQSRRSRRSVVE
137	-	FECV	Feces	2010	IA	TSSRRSRRSTPE
138	-	FECV	Feces	2010	IA	TQSRRSRRSVAE
140	-	FECV	Feces	2010	IA	TQSRRSRRSVVE
141	-	FECV	Feces	2010	IA	TQSRRSRRSVVE
142	-	FECV	Feces	2010	IA	THSRRARRSTVE
143	-	FECV	Feces	2010	IA	TSSRRARRSSVE
144	-	FECV	Feces	2010	IA	TQSRRSRRSASM
146	-	FECV	Feces	2010	CA	THSRRARRSTVE
149	-	FECV	Feces	2010	NY	TSSRRSRRSTPE
150	-	FECV	Feces	2010	NY	TSSRRSRRSTTE
152	-	FECV	Feces	2010	MI	THSRRSRRSNSD
153	-	FECV	Feces	2010	NY	PHSRRSRRSTNY
155	-	FECV	Feces	2010	NY	THSGRSRRSASD
167	-	FECV	Feces	2010	RI	THSKRSRRSTSN
4594	-	FECV	Feces	2004	MD	TQQRRSRRSTSD
4582	-	FECV	Feces	2004	MD	TQQRRSRRSTSD
D06 327	1	FIPV	Spleen	2006	NY	THSRRSRGSAPN
D06 327	2	FIPV	Mesentery	2006	NY	THSRRSRGSAPN
D06 244	1	FIPV	Mesenteric lymph node	2006	MI	TQSRRASTSTSN
D06 244	2	FIPV	Mesentery	2006	MI	TQSRRASTSTSN
D05 77	1	FIPV	Kidney	2005	NC	THSRRSRMSTQN
D05 77	2	FIPV	Cerebellum	2005	NC	THSRRSLRSTQN
07 129308	1	FIPV	Mesentery	2007	NY	TSSRRSPRSTLD
08 153990	1	FIPV	Kidney	2008	PA	TQPRRARMSVPE
08 153990	2	FIPV	Brain stem	2008	PA	TQPRRAPMSVPE
08 153990	3	FIPV	Cerebrum	2008	PA	TQPRRARMSVPE
08 153990	4	FIPV	Cerebellum	2008	PA	TQPRRARMSVPE
N05 48	1	FIPV	Cerebellum	2005	VA	TQSRSSRRSTSD
N05 110	1	FIPV	Mesenteric lymph node	2005	NY	TQTKRSRRSTPQ
N05 110	2	FIPV	Cerebellum	2005	NY	TQTKRSRRSTPA
N07 95	1	FIPV	Cerebrum	2007	NY	THTRKTRRSIAD
D04 397	1	FIPV	Spleen	2004	PA	TQSRRSRRSTVD
D04 397	2	FIPV	Mesenteric lymph node	2004	PA	TQSRRSRRLTSN
D04 93	1	FIPV	Kidney	2004	PA	THLRRSHRSTSE
D04 93	2	FIPV	Cerebrum	2004	PA	TLTGRSHRSTSE
151643	1	FIPV	Heart	2008	NY	TQFRRARRSAVR
151643	2	FIPV	Spleen	2008	NY	TQFRRSRRSTPG
151643	3	FIPV	Liver	2008	NY	TQFRRSRRSTVR
234	t1	FECV	Feces	2008	PA	THTRRSRRSAPV

Cat no.	Sample no.	FECV/FIPV	Sample Type	Year	State	Sequence
234	t2	FIPV	Kidney	2011	PA	THTRRSLRSAPV
304	t1	FECV	Feces	2008	PA	THTRRSRRSAPV
304	t2	FECV	Feces	2012	PA	THTRRARRSAPV

Technical Appendix Table 2. Primers used for reverse transcription PCR amplification of the feline coronavirus spike protein S1/S2 region to detect mutation in feline coronavirus

Primer	Nucleotide sequence (5′→3′)
FFPE Fwd2	GCACAAGCAGCTGTGATTA
FFPE Rev2 homology	GTAATAGAATTGTGGCAT
442(F)	GGCAGAGATGGATCTATTTTTGTTA
Sero1rev2a(R)	ATAATCATCATCAACAGTGCC

Technical Appendix Table 3. Fluorogenic peptides used in the furin cleavage assay to detect genetic mutation in feline coronavirus*

Peptide	Amino acid sequence
Canonical	THTRRSRRSAPA
P1′ S-L	THTRRSRRLAPA
P1 R-G	THTRRSRGSAPA
P1 R-M	THTRRSRMSAPA
P1 R-T	THTRRSRTSAPA
P2 R-H	THTRRSHRSAPA
P2 R-L	THTRRSLRSAPA
P2 R-P	THTRRSPRSAPA
P2 R-S	THTRRSSRSAPA
P3 S-A	THTRRARRSAPA
P4 R-K	THTRKSRRSAPA
P4 R-S	THTRSSRRSAPA
P5 R-K	THTKRSRRSAPA
P6 T-F	THFRRSRRSAPA
P7 H-Q	TQTRRSRRSAPA
P7 H-Q P5 R-K	TQTKRSRRSAPA

*All peptides contain a methylcoumarin acetamido/2,4-Dinitrophenyl fluorescence resonance energy transfer pair.

Technical Appendix Table 4. European Nucleotide Archive accession numbers for submitted sequences of feline coronavirus spike protein S1/S2 junction site

Cat-sample no.	Sequence	Accession no.
106	TQSRRSRRSYPD	HF954926
110	TQTRRSRRSTSE	HF954927
111	THSRRARRSTVE	HF954928
125	TQSRRARRSQFE	HF954929
126	TQSRRSRRSASS	HF954930
128	THSRRARRSTVE	HF954931
129	TQSRRSRRSTSD	HF954932
131	TQSRRSRRSASN	HF954933
132	TQSRRSRRSAPE	HF954934
135	TQSRRARRSLPA	HF954935
136	TQSRRSRRSVVE	HF954936
137	TSSRRSRRSTPE	HF954937
138	TQSRRSRRSVAE	HF954938
140	TQSRRSRRSVVE	HF954939
141	TQSRRSRRSVVE	HF954940
142	THSRRARRSTVE	HF954941
143	TSSRRARRSSVE	HF954942
144	TQSRRSRRSASM	HF954943
146	THSRRARRSTVE	HF954944
149	TSSRRSRRSTPE	HF954945
150	TSSRRSRRSTTE	HF954946
152	THSRRSRRSNSD	HF954947
153	PHSRRSRRSTNY	HF954948
155	THSGRSRRSASD	HF954949
167	THSKRSRRSTSN	HF954950
D06 327-1	THSRRSRGSAPN	HF954951
D06 327-2	THSRRSRGSAPN	HF954952
D06 244-1	TQSRRASTSTSN	HF954953
D06 244-2	TQSRRASTSTSN	HF954954
D05 77-1	THSRRSRMSTQN	HF954955
D05 77-2	THSRRSLRSTQN	HF954956
07 129308-1	TSSRRSPRSTLD	HF954957
08 153990-1	TQPRRARMSVPE	HF954958
08 153990-2	TQPRRAPMSVPE	HF954959
08 153990-3	TQPRRARMSVPE	HF954960
08 153990-4	TQPRRARMSVPE	HF954961
N05 48-1	TQSRSSRRSTSD	HF954962
N05 110-1	TQTKRSRRSTPQ	HF954963
N05 110-2	TQTKRSRRSTPA	HF954964
N07 95-1	THTRKTRRSIAD	HF954965
D04 397-1	TQSRRSRRSTVD	HF954966
D04 397-2	TQSRRSRRLTSN	HF954967
D04 93-1	THLRRSHRSTSE	HF954968
D04 93-2	TLTGRSHRSTSE	HF954969
151643-1	TQFRRARRSAVR	HF954970
151643-2	TQFRRSRRSTPG	HF954971
234-t1	THTRRSRRSAPV	HF954972
234-t2	THTRRSLRSAPV	HF954973
304-t1	THTRRSRRSAPV	HF954974
304-t2	THTRRARRSAPV	HF954975

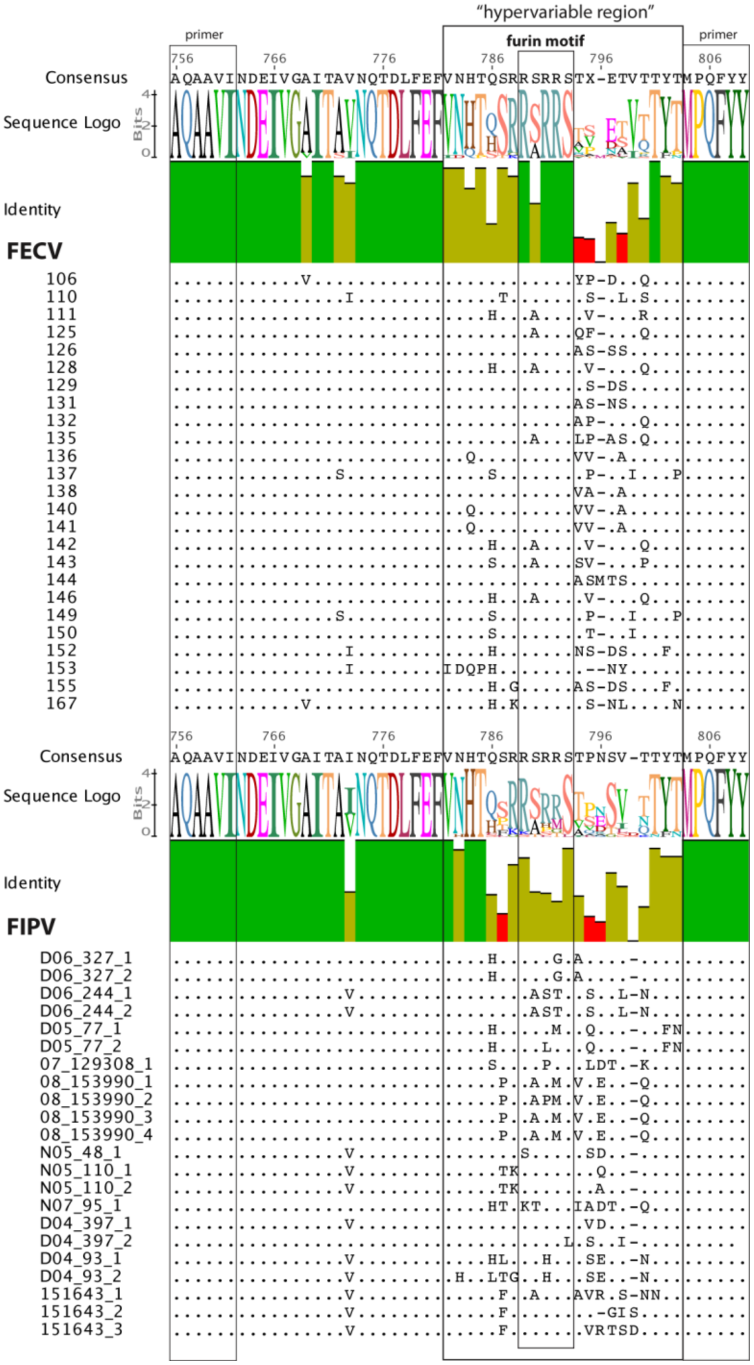

Technical Appendix Figure 1. Multiple sequence alignment of regions of FECV and FIPV spike sequenced in this study. Complete nucleotide sequences were translated and aligned by using Geneious version RG (Biomatters Ltd). Logo y-axis bars indicate the sequence conservation at each site, expressed in bits. A consensus, sequence logo, identity histogram, and alignment are displayed for the FECV and FIPV datasets. The primer annealing regions are boxed. There is moderate to high conservation of the consensus sequence outside of a hypervariable region, which spans from position

782 to position 802. It is within this hypervariable region that the conserved FECV furin motif RS/ARRS is found.

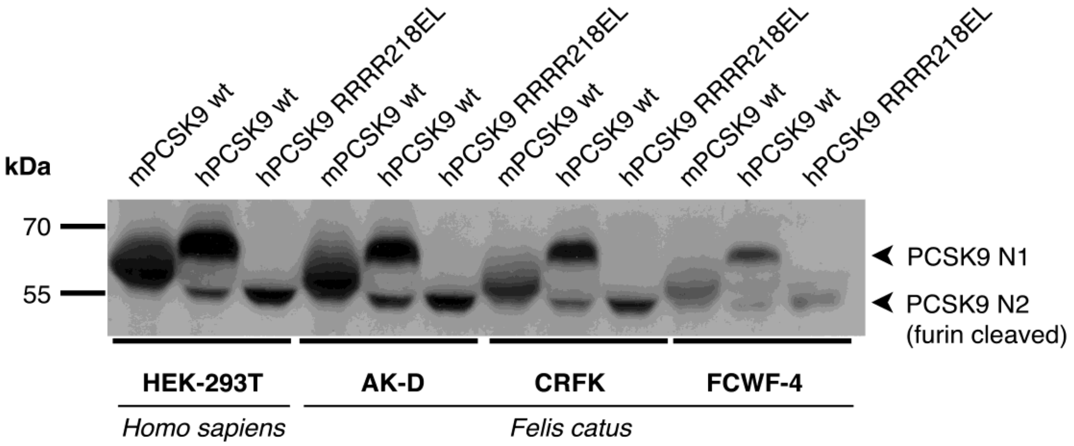

Technical Appendix Figure 2. Feline furin can proteolytically process PCSK9, a known substrate of human furin. Human HEK-293T, and feline cell lines AK-D, CRFK, and FCWF-4 were transfected with the wild-type (wt) mouse (m), wt human (h) and a human mutant form of PCSK9 (RRRR218EL) containing a furin canonical cleavage site, enabling complete cleavage by furin. All constructs contain a C-terminal V-5 tag. After undergoing proteolytic processing by several proteases, including furin, PCSK9 is secreted in the extracellular compartment. Twenty-four hours post-transfection, the supernatant of transfected cells were harvested and subjected to Western blot analysis and detection by using mouse monoclonal anti-V5 antibodies. The ~55 kDa (N2) band of the protein corresponds to the furin-cleaved form of PCSK9.
